# Multilevel Predictors of Dementia Progression in Mild Cognitive Impairment: A Nationwide Case-Control Study

**DOI:** 10.1093/geroni/igaf122.3626

**Published:** 2025-12-31

**Authors:** Song Hee Hong, Hayun Lim

**Affiliations:** Seoul National University, Seoul, Republic of Korea; Seoul National University, Seoul, Republic of Korea

## Abstract

**Introduction:**

Mild cognitive impairment (MCI) represents an early stage of cognitive decline with a high risk of progression to dementia. Since no effective drug treatments exist for dementia, identifying modifiable risk factors is critical. This study examined individual- and regional-level factors associated with dementia diagnosis among patients with MCI.

**Methods:**

A nested case-control study was conducted using nationwide health insurance claims data. The cohort included patients first diagnosed with MCI in 2017. Cases were individuals newly diagnosed with dementia in 2022 (n = 1,344), while controls were MCI patients without dementia (n = 5,376), matched 1:4 by age and sex. Individual-level variables included demographics, comorbidities, lifestyle, and medication use. Regional-level variables encompassed demographic aging indices, healthcare resources, and elderly welfare facilities. Conditional multivariate logistic regression was applied to individual-level factors, and multilevel logistic regression assessed regional-level influences.

**Results:**

Significant individual-level predictors of dementia included type 2 diabetes (OR = 1.14, 95%CI=1.00–1.31), depression (OR = 1.64, 95%CI=1.43–1.88), Parkinson’s disease (OR = 2.49, 95%CI=1.79–3.47), and hospitalization days (OR = 1.03, 95%CI=1.02–1.05). Protective factors included dyslipidemia (OR = 0.79, 95%CI=0.69–0.90), moderate physical activity (OR = 0.76, 95%CI=0.66–0.87), and adherence to dementia medications (OR = 0.71, 95%CI=0.58–0.87). At the regional level, a higher proportion of older adults increased dementia risk (OR = 1.01, 95%CI=1.00–1.02), while greater availability of elderly residential welfare facilities was protective (OR = 0.96, 95%CI=0.92–0.99).

**Conclusion:**

Both individual and regional factors significantly influence dementia risk among MCI patients. These findings underscore the need for integrated prevention strategies that combine clinical risk management with community-level resource planning to delay dementia onset and reduce its societal burden.

